# Systemic Inflammatory Patterns in Ovarian Cancer Patients: Analysis of Cytokines, Chemokines, and Microparticles

**DOI:** 10.1055/s-0043-1772590

**Published:** 2023-12-23

**Authors:** Aline Evangelista Santiago, Sálua Oliveira Calil de Paula, Andréa Teixeira de Carvalho, Eduardo Batista Cândido, Rafaela de Souza Furtado, Agnaldo Lopes da Silva Filho

**Affiliations:** 1 Department of Gynecology and Obstetrics, Faculty of Medicine, Universidade Estadual Paulista “Júlio de Mesquita Filho”, Botucatu, SP, Brasil; 2 Instituto René Rachou – Fiocruz Minas, Belo Horizonte, MG, Brasil; 3 Department of Gynecology and Obstetrics, Faculty of Medicine, Universidade Federal de Minas Gerais, Belo Horizonte, MG, Brasil

**Keywords:** cytokines, chemokines, microparticles, ovarian cancer, inflammation, citocinas, quimiocinas, micropartículas, câncer do ovário, inflamação

## Abstract

**Objective** To compare the patterns of systemic inflammatory response in women with epithelial ovarian cancer (EOC) or no evidence of malignant disease, as well as to evaluate the profile of systemic inflammatory responses in type-1 and type-2 tumors. This is a non-invasive and indirect way to assess both tumor activity and the role of the inflammatory pattern during pro- and antitumor responses.

**Materials and Methods** We performed a prospective evaluation of 56 patients: 30 women without evidence of malignant disease and 26 women with EOC. The plasma quantification of cytokines, chemokines, and microparticles (MPs) was performed using flow cytometry.

**Results** Plasma levels of proinflammatory cytokines interleukin-12 (IL12), interleukin-6 (IL-6), tumor necrosis factor alpha (TNF-α) interleukin-1 beta (IL-1β), and interleukin-10 (IL-10), and C-X-C motif chemokine ligand 9 (CXCL-9) and C-X-C motif chemokine ligand 10 (CXCL-10) were significantly higher in patients with EOC than in those in the control group. Plasma levels of cytokine interleukin-17A (IL-17A) and MPs derived from endothelial cells were lower in patients with EOC than in the control group. The frequency of leukocytes and MPs derived from endothelial cells was higher in type-2 tumors than in those without malignancy. We observed an expressive number of inflammatory/regulatory cytokines and chemokines in the cases of EOC, as well as negative and positive correlations involving them, which leads to a higher complexity of these networks.

**Conclusion** The present study showed that, through the development of networks consisting of cytokines, chemokines, and MPs, there is a greater systemic inflammatory response in patients with EOC and a more complex correlation of these biomarkers in type-2 tumors.

## Resumo

**Objetivo** Comparar os padrões de resposta inflamatória sistêmica em mulheres com câncer epitelial de ovário (CEO) ou sem evidência de doença maligna, bem como avaliar o perfil de respostas inflamatórias sistêmicas em tumores dos tipos 1 e 2. Esta é uma forma não invasiva e indireta de avaliar tanto a atividade tumoral quanto o papel do padrão inflamatório durante as respostas pró- e antitumorais.

**Métodos** Ao todo, 56 pacientes foram avaliados prospectivamente: 30 mulheres sem evidência de doença maligna e 26 mulheres com CEO. A quantificação plasmática de citocinas, quimiocinas e micropartículas (MPs) foi realizada por citometria de fluxo.

**Resultados** Os níveis plasmáticos das citocinas pró-inflamatórias interleucina-12 (IL12), interleucina-6 (IL-6), fator de necrose tumoral alfa ( *tumor necrosis factor alpha* , TNF-α, em inglês), interleucina-1 beta (IL-1β), e interleucina-10 (IL-10), e da quimiocina de motivo C-X-C 9 (CXCL-9) e da quimiocina de motivo C-X-C 10 (CXCL-10) foram significativamente maiores em pacientes com EOC do que nos controles. Os níveis plasmáticos da citocina interleucina-17A (IL17A) e MPs derivados de células endoteliais foram menores em pacientes com CEO do que no grupo de controle. A frequência de leucócitos e de MPs derivadas de células endoteliais foi maior nos tumores de tipo 2 do que naqueles sem malignidade. Observou-se um número expressivo de citocinas e quimiocinas inflamatórias/regulatórias nos casos de CEO, além de correlações negativas e positivas entre elas, o que leva a uma maior complexidade dessas redes.

**Conclusão** Este estudo mostrou que, por meio da construção de redes compostas por citocinas, quimiocinas e MPs, há maior resposta inflamatória sistêmica em pacientes com CEO e correlação mais complexa desses biomarcadores em tumores de tipo 2.

## Introduction

Epithelial ovarian cancer (EOC) is the fifth most frequent cause of cancer-related death in women, with an approximate yearly mortality rate of 7.0 per 100 thousand women. Most diagnoses are made when the disease is in an advanced stage (III or IV), which implies a five-year survival rate lower than 30%. 1 Epithelial ovarian cancer comprises a heterogeneous group of tumors, subdivided according to histological differences, by the degree of proliferation, and considering epithelial invasion. A dualistic model has been proposed, and EOC has been divided into types 1 and 2, depending on the histological, immunohistochemical, and molecular characteristics of the tumor. 2 Type-1 tumors are considered low-grade and usually originate from mutations on the *KRAS* , *BRAF* , *ERBB2* , *CTNNB1* , *PTEN* , and *PIK3CA* genes. Since they are genetically stable, they are less aggressive, which leads to a more favorable prognosis. In contrast, type-2 tumors are high-grade and have more uncontrolled cell differentiation, which culminates in an aggressive behavior. This is why they are usually diagnosed at an advanced stage and have a less favorable prognosis. They show TP53 mutations in more than 80% of the cases and repair DNA damage. A recent study 1 showed better disease-specific survival in type-1 than that in type-2 tumors, as well as the importance of the stage of the disease at the time of diagnosis in determining the survival rate.

Understanding the carcinogenesis of EOC is very important to determine the mechanisms involved in the origin and pathogenesis of these tumors. 3 The molecular biology of oncogenesis in ovarian cancer consists of multiple complex pathways, and previous studies 4 on the identification of prognostic markers for EOC have not yielded definitive results. There is growing evidence that an inflammatory process contributes to the growth of ovarian tumors and metastases to the peritoneum. 5 Therefore, the present study focused on ovarian cancer carcinogenesis and the role of inflammatory infiltrates in tumor progression.

Inflammatory mediators and various cytokines produced by the activated innate immune cells, such as tumor necrosis factor alpha (TNF-α), interleukin-1 beta (IL-1β) and proinflammatory cytokine interleukin-6 (IL-6), have been shown to promote the genesis, growth, and progression of EOC, with IL-6 being considered a central immunoregulatory cytokine. 6 This cytokine activates signaling pathways that lead to tumor-cell proliferation, and it appears to be involved in the process of tumor metastasis. 7 8 9 There is also evidence that cytokines and their regulators participate both in the process of ovarian cancer progression and in the chemoresistance of neoplastic cells, with IL-6 being one of the main immunoregulatory cytokines involved in this process. 7 8 Although the factors that regulate the activity of these cytokines in ovarian cancer are being studied, they are still unknown. 10 Regarding chemokines, C-C motif chemokine ligand 2 (CCL2) and C-C motif chemokine ligand 5 (CCL5), for example, are well recognized due to their activities in the immune context, stimulating the migration mainly of monocytes and T-cells to damaged or infected sites. 11

Microparticles (MPs) are a group of heterogeneous membranous vesicles with different shapes and sizes (ranging from 0.1 μm to 1 μm) called microvesicles (MVs). They are released from the cell membrane by the budding process of the external membrane, and determine similarities between MPs and their source cells, including the contents of substances of the mother cell, such as chemokines and cytokines, as well as genetic information to carry messenger RNA (mRNA), microRNA (miRNA), and genomic DNA. 11 12 This ability to incorporate components of the original cell and bring them to the recipient cells characterizes the importance of MPs in the process of intercellular communication, causing them to participate in several stages of cancer progression and resistance, such as metastasis, tumor angiogenesis, development of drug resistance, and evasion of immune surveillance. This, along with the fact that their molecules are promising biomarkers for the diagnosis, prognosis, and follow-up of the disease, makes MPs a great research subject. 11 13 14 15 16 Measuring the plasma concentrations of cytokines, chemokines, and MPs in women with cancer is a noninvasive and indirect way to assess tumor activity and the associated inflammatory/regulatory systemic response during the pro- and antitumor responses of the host. In addition, these molecules may also serve as biomarkers of disease activity and be used to monitor the treatment. In that regard, the aim of the present study was to compare the patterns of systemic inflammatory response in women with EOC and with no evidence of a malignant disease, as well as to evaluate the profile of the systemic inflammatory responses for tumor types 1 and 2.

## Materials and Methods

In the present study, we performed a prospective evaluation of 56 patients: 30 women with no evidence of malignant disease and 26 women with EOC. The study was approved by the Ethics Committee of Universidade Estadual Paulista “Júlio de Mesquita Filho” and by Hospital Vera Cruz Hospital, and all participants provided signed informed consent. The patients answered a questionnaire that encompassed many clinical and epidemiological variables, while the remaining clinical data were obtained from their medical records. The study included a control group composed of women with no evidence of malignancy or gynecological diseases and a second group of patients with EOC who underwent debulking surgery. The exclusion criteria for both groups were as follows: previous chemotherapy and/or radiotherapy; diagnosis of diseases of the immune system and/or use of corticosteroids or immunosuppressive drugs within the past six months, presence of any acute infectious processes in a laparotomy, and identification of a distinct EOC-related malignancy in the histopathological examination of the surgical specimen. In the EOC group, histological grading and disease staging were based on the International Federation of Gynecology and Obstetrics (Fédération Internationale de Gynécologie et d'Obstétrique, FIGO, in French) classification. In this study, ovarian cancer in FIGO stages I and II and FIGO stages III and IV were considered early and advanced diseases respectively. Only malignant epithelial tumors were included in the study, and borderline tumors were excluded. To distinguish between type-1 and type-2 tumors, the histological classification was not considered as the only parameter; we used clinical, histological, and immunohistochemical parameters. Type-1 tumors are diagnosed at early stages (I and II) with p53-negative immunohistochemical staining and classified as low-grade. Type-2 tumors are diagnosed in advanced stages (III and IV) with p53-positive immunohistochemical staining and classified as high-grade tumors. The histological subtypes included were endometrioid, clear-cell, mucinous, low- and high-grade serous, low- and high-grade adenocarcinoma/not otherwise specified, undifferentiated, carcinosarcoma, and granulosa-cell tumors. All cases were reviewed by a pathologist experienced in gynecological oncology.

### Purification of Plasma MPs

Flow cytometry was used to quantify the MPs in the plasma. Centrifugation of the citrated (0.5 mL) blood was performed at 1,500 × g for 15 minutes. Afterwards, the plasma was cooled to -20°C prior to storage at -80°C. The samples were then subjected to centrifugation at 13,000 × g for 3 minutes to obtain platelet-free plasma. This plasma was diluted (1:3) in phosphate-buffered saline (PBS) with citrate containing heparin and centrifuged at 14,000 × g for 90 minutes at 15°C. The resulting MP pellet was then resuspended in 1X annexin V (BD Biosciences, San Jose, CA, Unite States).

### Detection of Plasma MPs

Unless otherwise stated, all reagents and monoclonal antibodies (mAbs) used in the flow cytometry assays were obtained from BD Biosciences. The MPs isolated from plasma were gated (R1) based on their forward scatter (FSC) and side scatter (SSC) distribution in a density plot compared with the distribution of synthetic 0.7 μm to 0.9 μm SPHERO Amino Fluorescent Particles (Spherotech Inc., Libertyville, IL, United States). Considering the presence of the phosphatidylserine (PS) residues in the MP surface, the events present in R1 were assessed for positive annexin V staining (BD Biosciences), a classic microparticle marker, using mAbs conjugated with phycoerythrin (PE). Mouse immunoglobulin G (IgG) PE-conjugated isotype control mAbs were used to properly place the gates. Annexin V+ events gated in the R2 region were further assessed for immunolabeling with mAbs conjugated with fluorescein isothiocyanate (FITC) against cell markers CD66 (neutrophils), CD41a (platelets), CD51 (endothelial cells), CD235a (erythrocytes), CD45 (leukocytes), CD3 (lymphocytes), and CD14 (monocytes), or the corresponding mouse IgG FITC-conjugated isotype control mAbs. The samples were analyzed using a FACSCalibur flow cytometer (BD Biosciences). More than 100 thousand events were acquired for each sample, with at least 2 thousand events within the MP gate.

### Assessing Plasma Cytokine/Chemokine Levels using a Cytometric Bead Array Immunoassay

Analyzing secreted cytokine/chemokine with flow cytometry enables the simultaneous measurement of multiple biomarkers in a single sample. 12 17 18 To measure plasma biomarkers, whole blood samples were collected using ethylenediaminetetraacetic acid (EDTA) as the anticoagulant. Plasma was maintained at -80°C in aliquots and thawed just before use. The Cytometric Bead Array (CBA) immunoassay kit (BD Biosciences) was used for the quantitative analysis of the plasma biomarker levels. The CBA kit uses 7.5-μm polystyrene microbeads, distinct populations of beads that are unique due to their type-3 fluorescence intensity (fluorescence channel 3, FL- 3). Each bead is coupled to a biomarker-specific mAb, such as IL-1, IL-2, IL-6 IL-10, IL-12, IL-17a, TNF, interferon-gamma (IFN-γ), C-X-C motif chemokines ligands 8 , 9 and 10 (CXCL-8, CXCL-9, and CXCL-10), CCL- 2, and CCL-5, to capture the amount of protein detected in a direct immunoassay using a cocktail of different mAbs coupled to PE (fluorescence channel 2, FL-2). Briefly, 25 μL of plasma or standard (previously diluted in diluent G, as recommended by the manufacturer) were added to 15 μL of a bead cocktail and incubated for 90 minutes at room temperature in the dark. A biomarker standard calibrator mixture was used for each assay. After incubation, both the samples and standards were washed with 500 μL of wash buffer (supplied with the CBA kit) and centrifuged at 600 × g for 7 minutes at room temperature. Subsequently, 20 μL of detection cocktail – consisting of six PE-conjugated mAbs – were added to each tube, and the mixture was reincubated for 90 minutes at room temperature in the dark. Following incubation, the samples and standards were washed again with 500μL of wash buffer and centrifuged at 600 × g for 7 minutes at room temperature to remove the unbound detector reagent. After washing the samples, 250 μL of wash buffer were added to each tube prior to data acquisition using a fluorescence-activated cell sorter (FACS) Calibur flow cytometer (BD Biosciences). Although the fluorescently-labeled particles in the BD CBA immunoassay are designed to be excited by the 488-nm laser that is commonly found on all BD flow cytometers, they can also be excited by the red diode laser on dual-laser BD FACS Calibur instruments. The detection of particle emission on fluorescence channel 4 (FL-4) simplifies instrument setup and requires less fluorescence compensation. Thus, a total of 1,800 events/gate were acquired once the flow cytometer was properly setup to measure the FSC and SSC. Dual-color (FL-4 and FL-2) flow cytometric acquisition using a dual-laser BD CBA template was also conducted. Data analysis was performed using the BD Biosciences CBA software. The results were expressed in pg/mL.

### Statistical Analyses

The Mann–Whitney test was used in the comparison between the groups and variables of interest. 18 The software used in the analyses was R (R Foundation for Statistical Computing, Vienna, Austria), version 3.5.2. Non-normal variables were expressed as median and interquartile range (IQR, 25 ^th^ –75 ^th^ percentiles) values. Correlations were analyzed using the Spearman two-sided test and the GraphPad Prism (GraphPad Software, Inc., San Diego, CA, United States) software, version 5.00. Values of *p* < 0.05 were considered statistically significant.

### Network Analyses

The Spearman correlation test was used to assess the correlations involving biomarker levels. The correlations were classified as negative or positive, and the correlation index (r) was used to categorize the correlation as weak (r < 0.35), moderate (r ranging from 0.36 to 0.67), or strong (r > 0.68). Then, networks of biomolecular interactions were developed to evaluate the correlations among cytokines, chemokines, and MPs for each clinical group using the Cytoscape (open source) software, version 3.0.2. 19 Tables involving the characteristics of the correlations (type and strength) and the different parameters to be correlated (cytokines, chemokines, and MPs) were created. These tables were then imported to the Cytoscape software and built into the networks, in which the nodes represented the source and target interactions (cytokines, chemokines, and MPs) determined in the imported table. Dotted lines represented negative correlations and solid lines represented positive ones. The strength of the correlation was represented by the thickness of the lines: the thicker the line, the stronger the correlation. The positive and negative correlations were significant when *p* < 0.005.

## Results

**Fig. 1:**
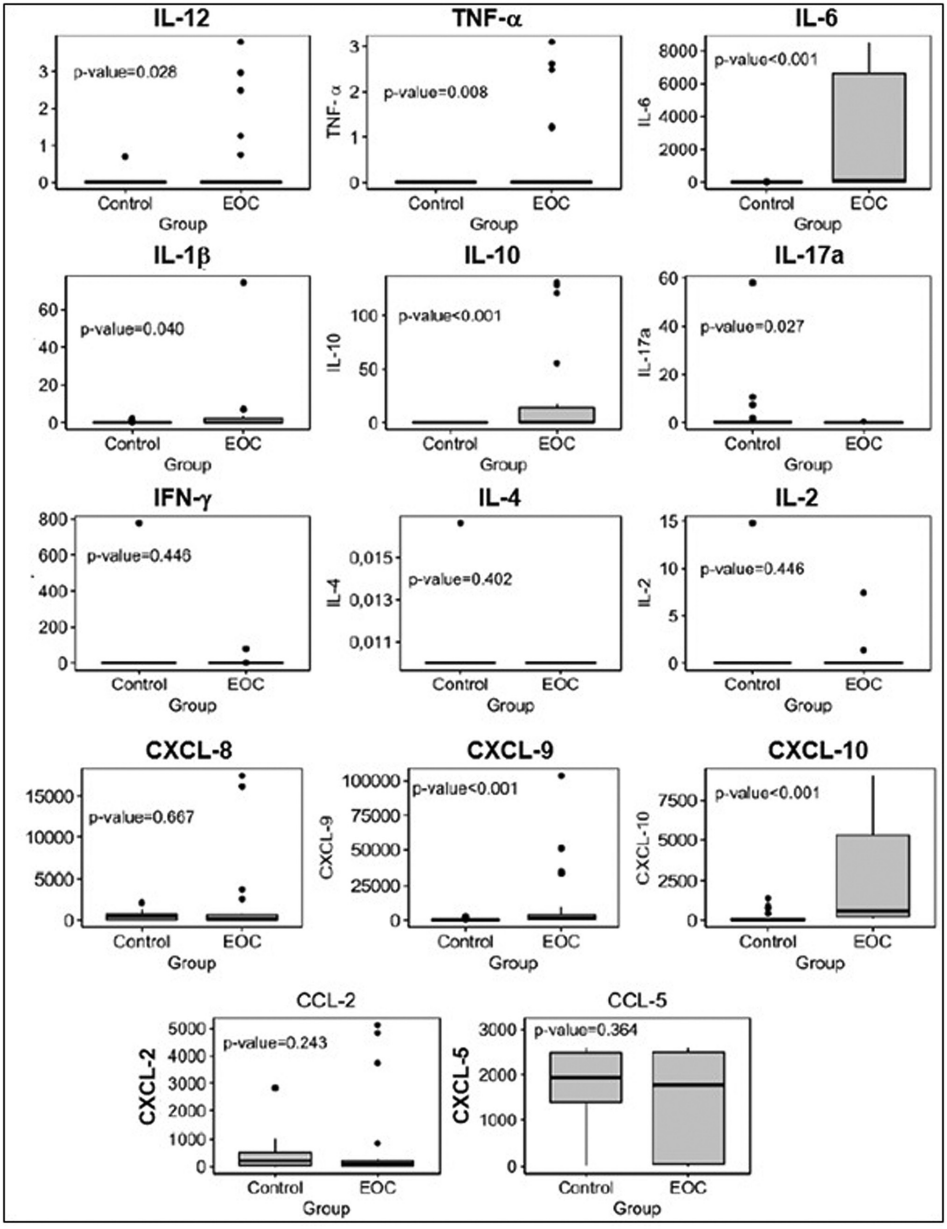
Boxplot – plasma biomarkers (pg/mL). Comparison of proinflammatory cytokines (IL-1β, IL-6, TNF-α IL-12, and IFN-γ), regulatory cytokines (IL-2, IL-10, and IL-17a), and chemokines (CCL-2, CCL5, CXCL8, CXCL9, and CXCL10) in the control group and in ovarian cancer patients. Abbreviation: EOC, epithelial ovarian cancer. Notes: Data were expressed as median with interquartile range values. Differences between groups were evaluated using the Kruskal-Wallis test.

**Fig. 2:**
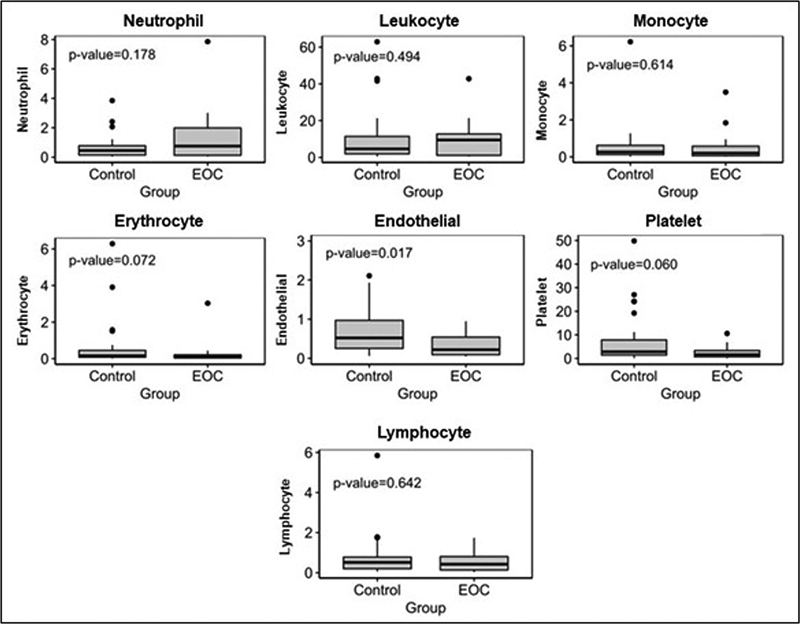
Boxplot – circulating microparticles. Comparison of the circulating microparticles (MPs) between the control group and ovarian cancer patients according to the specific cellular origin. Abbreviation: EOC, epithelial ovarian cancer. Notes: Data were expressed as median and interquartile range values. Differences between groups were evaluated using the Kruskal-Wallis test.

The mean ages of the control group and EOC patients were of 55.8 ± 6.8 years and 62.3 ± 14.1 years respectively ( *p* = 0.497). In total, 10 (38.5%) of the EOC patients had stage-I/II ovarian cancer, and 16 (61.5%) had stage-III/IV ovarian cancer. Among them, 8 (30.8%) had type-1 tumors (7 in stage III and 1 in stage III/IV), and 18 (69.2%) had type-2 tumors (3 in stage III and 15 in stage III/IV), and patients with type-2 EOC were in more advanced stages compared with those with type-1 tumors ( *p* = 0.001). Figs. 1 and 2 present the description of the variables of interest for the groups. Fig. 1 showed plasma levels of proinflammatory cytokines IL12 ( *p* = 0.028), IL-6 ( *p* < 0.001), TNF-α (= 0.008), IL-1β ( *p* = 0.04), and IL-10 ( *p* < 0.001), and chemokines CXCL- 9 ( *p* < 0.001) and CXCL-10 ( *p* < 0.001), which were significantly higher in the group of patients with EOC than in the control group. Another important difference between the groups was the lower level of cytokine IL-17a in the group of EOC patients ( *p* = 0.027).

This significant difference between the groups was also observed in relation to endothelial cell-derived MPs. Their levels were lower in EOC patients than in the control group ( *p* = 0.017) ( Fig. 2 ).

**Fig. 3:**
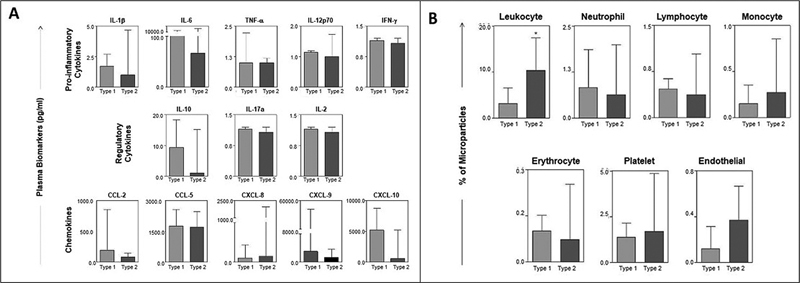
( **A** ) Levels of cytokines and chemokine in ovarian cancer patients according to tumor type. The results were presented in a column-chart format and were expressed as median and interquartile range values in pg/mL. Statistical differences were considered significant when *p* < 0.05. ( **B** ) Percentage of circulating MPs in patients with type-1 and type-2 tumors according to the specific cellular origin. The results were presented in a column-chart format and were expressed as the median and interquartile range values in pg/mL. Statistical differences were considered significant when *p* < 0.05.

The percentage of circulating cytokines, chemokines, and MPs in patients with type-1 and -2 tumors, according to their specific cellular origin, was evaluated and is shown in Fig. 3 . There were no differences in the plasma levels of cytokines and chemokines between type-1 and -2 tumors. However, the frequency of leukocytes and MPs derived from endothelial cells was higher in type-2 tumors than in those without malignancy ( *p* < 0.005).

**Fig. 4:**
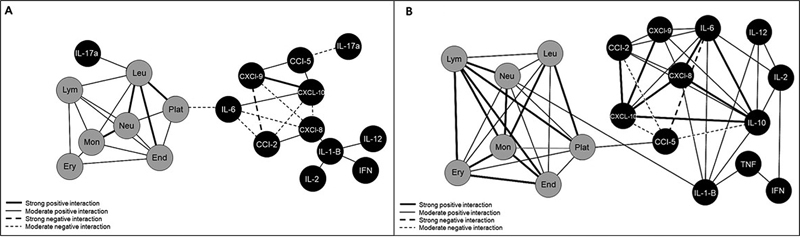
( **A** ) Biomarker networks in the control group. ( **B** ) Biomarker networks in EOC patients according to their specific cellular origin. Notes: MP nodes were assembled, and biomarker correlation indices were established between groups. The strength of the interactions was represented by different line styles according to the following ranges: negative (r < 0–dotted line), positive (r > 0–continuous line); weak (r ranging from 0 to 0.36–thinner line), moderate (r ranging from 0.36 to 0.67), and strong (r > 0.68–thicker line).

To evaluate potential relationships among cytokines, chemokines, and MPs in the EOC and control groups, all data obtained in the present study were used to develop the biological networks, in which the nodes represented the cytokines, chemokines, and MPs that were evaluated, and the lines represented positive or negative correlations and strong, weak, or moderate levels ( Fig. 4 ). It was possible to observe that there was a balance among cytokines, chemokines, and MPs in the control group, with fewer and weaker connections between the biomarkers. In EOC patients, the first cluster was characterized by strong and moderate correlations between the MPs, and the cytokine and chemokine networks were moderately correlated to the MP network. When comparing the network of the EOC patients to that of the control group, the former presented a larger number of inflammatory/regulatory cytokines and chemokines, as well as both negative and positive correlations between them, which led to a higher complexity of these networks.

**Fig. 5:**
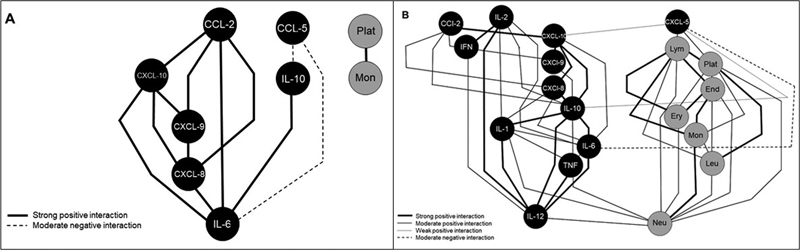
( **A** ) Biomarker networks in type-1 tumors. ( **B** ) Biomarker networks in type-2 tumors. Note: chemokine, cytokine, and MP nodes were assembled, as well as the biomarker correlation indices among groups (negative, moderate, and strong-positive correlation).

We also established a cellular interaction network between both types of tumors, observing many strong and moderate correlations involving cytokines, chemokines, and MPs, and only a negative interconnection in the networks. Additionally, type-2 tumors presented more correlations than type-1 tumors ( Fig. 5 ).

## Discussion

Ovarian cancer is a heterogeneous group of malignancies, and EOC is its most fatal type. Due to their non-specific symptoms, they are usually diagnosed at an advanced stage. To date, there are no reliable screening tests and diagnostic methods to detect the disease at an early stage. Therefore, studying the carcinogenesis of ovarian cancer and developing effective screening detection strategies to detect the disease in its early stages is of utmost importance. This is believed to be the best method to develop a successful treatment and ensure improved survival for patients with ovarian cancer. 19

In recent years, the role of cytokines in carcinogenesis and their participation in intercellular communication has been well established by several authors. 7 9 16 However, despite the known proinflammatory or regulatory effects of inflammation, it is unclear whether cytokines have any application in cancer treatment, especially in epithelial tumors. In 1996, a study 20 on ovarian tissue showed that it contained several proinflammatory growth factors, cytokines, and chemokines. Subsequently, other studies 21 22 23 showed a predominantly humoral immune response and an immunosuppressive pattern with IL-6, IL-10, and IFN-γ associated with EOC. In the present study, we investigated plasma cytokines, chemokines, and MP levels in both women with EOC and a control group. EOC patients showed higher levels of proinflammatory cytokines (IL-6, TNF-α, and IL-12), regulatory cytokines (IL-10), and chemokines (CXCL-9 and CXCL-10), which corroborated this environmental proinflammatory/regulatory mechanism for the development of ovarian cancer.

Some cytokines and chemokines have a protagonist role in literature, such as IL-6, whose signaling seems to play a leading role in the inflammatory process, and it is one of the major immunoregulatory cytokines found in the EOC microenvironment. Therefore, it has been proposed that IL-6 is a central cytokine that promotes ovarian cancer progression, although its exact role during disease development has not been well established. In ovarian cancer, IL-6 antagonist signaling has been accepted as having a therapeutic potential through inhibition of the cytokine network. 11 24 In another study, 25 which analyzed the level of cytokines in the peritoneal fluid of patients with ovarian cancer, higher levels of IL-6 were related to shorter disease-free survival and overall survival. In the present study, we evaluated this cytokine and found a significant difference in its expression in the EOC group when compared with the control group ( Fig. 2 ), highlighting its importance for ovarian cancer, and possibly for carcinogenesis.

In addition, IL-12 is known to increase the antitumor activity of natural killer (NK) cells, and its activity is antagonized primarily by IL-10, with its immunosuppressive or immunostimulatory action. 26 27 28 The present study showed that these cytokines not only played an important role in ovarian cancer, but that they also interacted in the process.. Further studies on this may lead to potential strategies against ovarian cancer.

Various types of cell secrete CXCL10, including endothelial cells stimulated by IFN-γ wh h h IL-12 cytokine family. 29 This revealed that, compared with the control group, the EOC patients presented increased levels of CXCL10 and decreased levels of MPs derived from endothelial cells, which could be explained by the increased activity of endothelial cells in ovarian cancer, and the related increased production of CXCL10 and lower release of microparticles.

In the present study, we found increased percentages of leukocyte-derived and endothelial-derived MPs in type-2 tumors compared with type-1 tumors, although endothelial-derived MPs were less frequent in the EOC group than in the control group. These results corroborate the dualistic model that categorizes EOC into two types, and suggest a difference in susceptibility to carcinogenesis in both tumors. In contrast, it does not provide an explanation for the decreased levels of endothelial-derived microparticles in the EOC group.

Interactions involving cytokines, chemokines, and MPs and their isolated effects have been reported in literature, 6 7 including those related to carcinogenesis. Among these are the correlations regarding proinflammatory cytokines IL-6 and TNF-α with the immunosuppressive cytokine IL-10 for a poor prognosis of EOC, the correlation between elevated levels of IFN-γ and increased survival, and the correlation between elevated levels of IL-6 and IL-10 with lower survival rates. 21 However, the complex network of interactions involving these structures has not been clearly elucidated, and a better understanding may lead to the development of potential cancer therapies. To better understand the correlations involving cytokines, chemokines, and MPs and, consequently, find possible diagnostic or prognostic tumor markers, hierarchical networks were used to simulate the inflammatory environment of the studied groups. Noteworthy, the supposed global relationships regarding cytokines, chemokines, and MPs were found in clusters. Using the Cytoscape software, we could create complex networks, which graphically showed the inflammatory profile of each group, as well as the correlations involving different parameters and the characteristics of each correlation. Women with ovarian malignancy presented a greater number of strong interactions between inflammatory and immune factors, especially ones involving CXCL-8, and greater complexity in all interactions. This may reflect a greater systemic inflammatory response in ovarian cancer and the involvement of a higher number of possible tumor markers and different interactions among them. This result can be explained by the specific location of ovarian epithelial cells in the peritoneal cavity, where they are clearly exposed to various proinflammatory agents. 30 These results are in agreement with those of studies 26 31 that show that, in EOC, there is a larger proinflammatory microenvironment and a more complex communication pathway, with the exchange of signaling factors that together can support tumor growth and progression. Another noteworthy finding was the substantial difference between networks from type-1 and type-2 tumors, with a greater number of correlations present in type-2 tumors. This result may reflect the discrepancies in the carcinogenesis of both tumors. Our data suggest that there is an interaction involving these soluble factors which are crucial for tumor growth and may validate this network as a key therapeutic target in ovarian cancer.

The present study was not limited to the quantification of cytokines, chemokines, and MPs to evaluate the inflammatory response involved in ovarian carcinogenesis. Interaction networks developed among these biomarkers demonstrated the greater complexity involved in the inflammatory response to EOC. Another strength of the present study is the molecular comparison of type-1 and type-2 EOCs, which shows a different pattern of interactions involving the biomarkers although it did not present quantitative differences between the groups. The present study had several limitations. The EOC group was not divided according to the tumor histology, which did not enable the analysis of the inflammatory response pattern in each histological type. In addition, the study had a limited number of patients, which reflected the low prevalence of the disease.

## Conclusion

The results of the present study enable us to conclude that there are different patterns of systemic inflammatory response assessed by the levels of cytokines, chemokines, and MPs in women with EOC and without evidence of malignancy, and a greater systemic inflammatory response in patients with EOC was observed. The study also showed that type-2 tumors present more complex correlations regarding these biomarkers than type-1 tumors. Since tumor markers are potential tools for screening, diagnosis, prognosis, and posttherapy follow-up in cancer treatment, these molecules are important targets for further studies. Additionally, this may lead to the prescription of specific types of targeted therapies to patients depending on the inflammatory response profile of their disease. Therefore, a full understanding of cancer immunobiology will stimulate the development of more effective immunotherapeutic approaches against these tumors.
